# House Building—From the Hygienic Point of View

**Published:** 1870-07

**Authors:** Ben H. Pratt


### THE BISTOURY.

VOL. VI. ELMIRA, N. Y. JULY, 1870. NO. 2.

### Communications.

### House Building—From the Hygienic Point of View.

BEN H. PRATT, A. M.

In our day, there is no known science, art, ism or ology without its appropriate organ, conducted by its special advocates, and supported by its special patrons and disciples.

By reason of its antiquity, utility, beauty and sublimity, Architecture, by universal consent, rears its head far above all arts and asserts its claim to a greater share of the light of modern wisdom than any other.

In conformity with this acknowledgment, the press daily teems with elaborately wrought disquisitions upon this grand theme. Whol periodicals are devoted to its interests. Vast cyclopediæ astound and bewilder us with their learned and erudite treatises upon its merits.—Stumbling along over its mystic technicalities—floundering through its musty antiques—utterly lost in the mazy labyrinths of its metaphysical literature—we come to a gap, a chasm, yawning, unbridged, scarcely essayed, to wit: the Hygiene of Architecture, or, Architecture in its relations to health. Strange as it may appear, this department of this grand old study has for ages gone uncared for, and even now gets but a tithe of its deserts.

But lest I may have seemed to err in episodically touching upon the more dignified topic of Architecture, while my paper bears only upon our ordinary house building, I will enter upon my subject—one vitally important to all, and especially so to the provincial reader and dweller.

That we may build well and tastefully—combine the practical with the æsthetic—is no more desirable nor prudent than that we may build healthfully. To assist those, then, who are willing and eager to combine beauty, utility and health in their dwellings, is the object of this article.

To the mind of the writer, the security of health in our dwellings depends upon three general principles—a healthful *location* and *position;* plenty of *sunshine;* plenty of *air*.

The majority of those whose eye this paper will meet, cannot always elect a location.—Where fortune has placed them in the attainment of the necessaries and comforts of life, thence it is not often practical to remove; but even in the few assuredly unhealthful districts of our land, there are degrees of excellence in this respect, and hence choices of location. In general, never build upon low ground, nor even upon an elevation that inclines toward the site in more than one direction. Nature singularly fashions cess-pools at unlooked-for altitudes sometimes. Having secured a location, let your foundation extend at least two feet above the highest ground; this, in order to secure sure circulation of air beneath floors. The hygienic effect of the cellar is not so universally appreciated as it should be, and ought to extend under the whole house. Thus uniformity of temperature is preserved upon the floor, and rendered warmer in winter and cooler in summer than without. Again, no surer breeder of miasmatic air exists than a tightly underpinned, cellarless house. If in doubt, attempt to burn a candle in such a place before admitting any considerable quantity of fresh air. Witness the premature decay of joists and flooring beneath such buildings, and believe. Cellar windows should be as numerous as those of the first story, and not the miserably few and narrow crevices we too frequently meet with. If the locality be subject to occasional floods, or inundations from swollen streams by percolation, the only cause of alarm is in the duration and not in the quantity of water. If it is soon absorbed by the earth after the cause subsides, no harm can come of a little water in one's cellar. If, however, it remains long after the rise which produced it is gone, you are unfortunate in your location and cannot dwell there with impunity. Trust not the vaunts of a mason whose cement will keep water *out*. Retaining water *within* a cemented excavation, as a cistern, is practicable; keeping it *out* is not. The philosophy of this is readily seen, and needs no elaboration.

The next desideratum is sunshine—plenty of it—great volumes of this life-giving, health-bringing blessing—this brightest, cheeriest, most exhilarating of God's good gifts to men. Therefore spare no pains nor expense in bewindowing your dwellings, even at the expense of looks. And let them be no sneaky little mortises either, but great, generous flood gates of healthful light. Those of the first floor should reach from base board to within a foot of the ceiling—those above should have their sills not more than two feet from the floor. Halls, entries, closets, store rooms, garrets, all should be lighted in some way, and amply; if not directly, then from some well lighted apartment. Guard jealously every hindrance to the free admission of daylight. For this reason, build, if possible, where no neighbor can run a wall between you and this blessing. Avoid houses in blocks, or that adjoin others. Cut down every tree that beglooms your windows, yet retain those which offer agreeable shade. Give every room a daily sunbath, and avoid the purchase of furniture or carpets so delicate that they can't stand any glory. Shun dark ceilings and leathery wall papers that are now so unfortunately fashionable. The dark woods of our modern furniture and the gloomy, somber style of most oil paintings, are all that our rooms will endure without lugging gloom upon gloom and converting our dwellings into dungeons.—Ugh! It makes one's blood chill and flesh creep to walk into some modern drawing room, and sit while the domestic summons the lord or lady of the gloom; and we mentally hum,

"In the prison cell I sit," &c.

From such a place for our little sun-loving Jacks and Jils, good Lord deliver us.

But, guilty as most are in a dereliction of duty in the matter of sunshine, guiltier are more in the remaining requisite to healthful building, ventilation. It is comparatively a late thing that any thought, much less effort has been devoted to this important item in the erection of dwellings. People have gone on for centuries, fashioning air-tight receptacles for themselves and families, as though a little fresh air would be their doom; or as if they were destined to be put up for preservation on the preserving can principle, making self-sealing jars of their houses, and canned sweetmeats of their inmates. Every Fall there was strawing of cellars—listing of doors—calking of all possible and impossible cracks—cottoning of keyholes, and kindred other asphyxiating processes. The idea of a draft entering the house was as much an object of terror as the small pox, or a book agent. This error is yet but partially overcome, and our dear grandmas still hear, with uplifted hands and horrified stare, that we are actually having our houses built with a view to perpetual drafts in every apartment.

To secure plenty of fresh air in our dwellings, it must come from out of doors, and so come as to keep up the supply as fast as the internal air is exhausted of its oxygenating or life-giving properties, and no faster. To secure this end has well nigh baffled the efforts of mankind; but at last an approximation to the true principle has been found, viz.: by admitting pure air at the upper part of the apartment, either by means of lowering upper sashes, or by ingeniously contrived air flues, and releasing foul air at the lower portion of the room. The well known fact that impure or carbonated air is heavier than ordinary atmosphere, and lies in strata near the floor has led to this method of ventilation, and the results of oft-repeated experiments bear out the correctness of the theory. A thorough plan is to build flues in the walls, accessible to every apartment in the house, and communicating with the outer air by any device one fancies, but generally opening at the roof, and concealed by a chimney-like finish or turrets which are often made highly ornamental. These must communicate with the room at the base board, or better still, on a level with the floor. The natural ingress of air upon the casual opening of doors and occasional lowering of upper window sashes, being pure, forces an equivalent volume of vitiated air out of the room by the flues before mentioned, no pure air escaping since it cannot come in contact with the opening.

Aside from this more expensive method of ventilation, a result scarcely less beneficial is secured by high ceilings and roomy apartments, with less punctitious notions about cracks, spaces between window sash and frame, etc., etc. Over every door in a prudently arranged house there should be a transum, or revolving window, and the heating apparatus should consist of fire places, grates, or heaters, whose cold air drafts enter from out of doors, rather than stoves, which simply re-heat the same air again and again until every particle of its vitalizing principle is destroyed.

A great error, but one that does not properly belong to my present subject, is the adoption of the modern low bedsteads, which keep us dipped in air saturated with carbonic acid gas all night. Unless the breather is above the window sill, and the window more or less open, he must breathe poisoned air. There is no help for it unless in the case of floor ventilation.

Too much importance cannot be attached to the benefit of having the sitting, family, or living room of ample dimensions. It should be the largest, airiest, cheeriest room in the whole house, capable of thorough ventilation, which a good grate, or an old fashioned wood fire (alas, we seldom greet the latter now,) will surely effect. Its windows should admit of being readily opened at top as well as bottom. Such a room will, of itself, predispose a family to enjoyment, since there is no more baneful foe to cheerfulness and communicativeness than the respiration of impure air.

The culpable negligence of those who build homes for themselves is never more fully exemplified than in the omission of a bathing room. It need not be an expensive nor an elaborate affair, and its effect upon health as well as comfort and cleanliness is inestimable. The baths we take in a wash bowl are sorry farces, and should never be depended upon for health or cleanliness, however thorough we may suppose them to be. O, the vigor, the elasticity, the rejuvenating effect of a cold plunge bath, with flesh-brush and crash towel accompaniment!

In a family of children, a play-room, where the youngsters may indulge themselves to repletion in noise and romps—where spoilable furniture is not, nor yet parental hushings oft—is the exception and not the rule with builders. Don't let your selfishness get the better of you in this matter. Give the little ones a show for their lungs and limbs on a rainy day. Remember you were once a child, and if you didn't have such a place, how you longed for its attainment. It pays—pays in strength—pays in health—pays in quiet—pays in furniture preserved—pays in fun—pays in a hundred ways, does the play room.

In a sketch of Hygienic House Building, brief as this must necessarily be, we cannot further enter into the merits of the subject; indeed what has been already said is of so general a character as to interfere with the conveyance of many pet ideas and theories which impress me as emgularly important and singularly overlooked. That what is said here may prove suggestive of many other needs, to the intelligent builder is all I dare hope for. Though nothing new nor strange has been offered, a consciousness of having broached a subject replete with benefit to mankind everywhere—a subject which merits, and ought to get, the attention of all who have their own and family's interests at heart—augurs an effect which cannot be bad and may prove beneficial.

There seems to be a feeling among those making homes for themselves, that everything is accomplished when they have created a proper looking and conveniently-arranged dwelling, quite as good as their neighbors', and embracing all the so called "modern improvements;" forgetful that the *summum bonum* of all comfort is health, and the acme of all misery the lack thereof. Big houses, built for show or company, may bring a certain sort of repute to the owner, but are a cruel care to delicate wives and mothers—in a word, unhealthful. Little boxes, yclept cosy residences, are smothering institutions, and sap and undermine the foundation of many a vigorous constitution. There is a happy mean which he will take who is wise, and health and its willing companion happiness will repay him a handsome precentage on his domestic investment. Builders of churches would do well to have more regard for ventilation and sunshine than for costly spires, curious gables, and turrets of no earthly (nor heavenly) use that we could ever learn, save to show what blind and foolish imitators we are of a barbaric age. Piety as well as other virtues, needs air and sunshine to render it of any practical worth.

---

To remove freckles, cut them out with a razor and throw them away. To bring out a moustache, tie it to a strong cord, twenty feet long, to the other end of which attach a heavy smoothing iron, and throw the latter from a fourth-story window. To get rid of red hair, hold your head for a few minutes in a strong blaze of gas. To preserve your eyes, put them in a bottle filled with alcohol. To avoid corpulence, quit eating. To conceal your teeth, keep your mouth shut. To keep out of debt, acquire the reputation of a rascal, and no one will trust you. These are infallible receipes.

